# Evaluation of the Impact of Cisplatin on Variances in the Expression Pattern of Leptin-Related Genes in Endometrial Cancer Cells

**DOI:** 10.3390/ijms21114135

**Published:** 2020-06-10

**Authors:** Dariusz Dąbruś, Robert Kiełbasiński, Beniamin Oskar Grabarek, Dariusz Boroń

**Affiliations:** 1 Faculty of Health Science, Public Higher Medical Professional School in Opole, 45-060 Opole, Poland; 2 Department of Obstetrics and Gynaecology ward, Health Center in Mikołów, 43-190 Mikołów, Poland; rkielbasinki111@gmail.com; 3 Department of Histology, Cytophysiology, and Embryology, Faculty of Medicine in Zabrze, the University of Technology in Katowice, 40-055 Katowice, Poland; bgrabarek7@gmail.com (B.O.G.); dariusz@boron.pl (D.B.); 4 Department of Clinical Trials, Maria Sklodowska-Curie National Research Institute of Oncology Krakow Branch, 31-115 Kraków, Poland; 5 Department of Gynecology and Obstetrics with Gynecologic Oncology, Ludwik Rydygier Memorial Specialized Hospital, 31-826 Kraków, Poland

**Keywords:** leptin, cisplatin, siRNA, supplementary molecular marker, apoptosis

## Abstract

This research aimed to assess the impact of cisplatin, depending on the concentration and exposure time, on the expression pattern of leptin in an endometrial cancer cell line. Ishikawa endometrial cancer cell cultures were incubated with cisplatin, at concentrations of 2.5–10 µM, or leptin in the concentration range 10–40 ng/mL, and for durations of 12, 24 and 48 h compared with the control. The microarray techniques: RTqPCR; ELISA; and RNAi assay were used. Statistical analysis was performed at *p* < 0.05. Already with the lowest concentration and incubation time, statistically substantial silencing of leptin expression on the mRNA level under the influence of cisplatin after its addition to the culture was observed. On the protein level, the expression for cisplatin at a concentration of 2.5 µM was only noticeable after 48 h of exposure and maintained themselves with consecutively larger concentrations. It was observed that cisplatin at a concentration of 5 µM is IC_50_ and the drug activated apoptosis via caspases -3 and -9. Cisplatin at a concentration of 5 µM and higher has a significant effect on the concentration of leptin. The effect of cisplatin on the expression profile of genes associated with leptin-dependent signaling pathways and changes in the expression of leptin itself and its receptors was confirmed. It was also confirmed that cisplatin exerted its effect via the leptin pathway.

## 1. Introduction

Leptin is a peptide hormone, showcasing an ability to interact with a specific leptin receptor (LEPR) receptor, which leads to the initiation of signaling pathways. Its physiological role is connected with the regulation of appetite and energy release [1,2]. Adipose cells or adipocytes were noted to have an expression of leptin, its expression was also noted in the placenta, stomach, skeletal muscles and the epithelium of the breasts [3]. Nonetheless, adipose tissue plays a key role in physiological processes, a disturbed expression level of leptin was observed in the course of cancer [4,5].

Cancerous cells are characterized by an ability to express on the surface of their specific receptor towards leptin and additionally towards the secretion of leptin, therefore, this indicates that the biological effects of leptin, in the case of cancerous diseases, are intensified. What is of significance, for example in the case of cancer of the large intestine, is that it is indicated that determining the level of leptin could be a promising molecular marker for tracking the progression of a tumor, as the concentration of leptin correlates with tumor cell proliferation and the neo-angiogenic process [6]. Substantially larger levels of leptin than the physiological level were observed in: bladder cancer; breast cancer; large B cell lymphoma; ovarian cancer; and testicular cancer, inter alia [7].

Sultana et al. based on their analyses showed that the increase in the expression level of leptin mRNA correlates with a worse prognosis in patients with ovarian cancer [8]. In turn, Wai et al. highlighted that increased expression of leptin was an unfavorable prognostic marker of survival among patients with ovarian cancer [9].

An improper level of leptin was also found in the course of endometrial cancer, a common gynecological cancer in women with menopause [10]. Cymbaluk et al. noted a substantially larger average concentration of leptin (*p* < 0.0001) in the serum of patients with endometrial cancer and endometrial hyperplasia on the level of 16,737.1 pg/mL vs. 9048.7 pg /mL in female patients without an established pathology within the endometrium (control) [11]. Furthermore, it is also valuable to note that leptin is indicated as a new, supplementary molecular marker of the neoplastic process, as well as a promising indicator for monitoring the effectiveness of pharmacotherapy. Moreover, it has been determined that a higher concentration of leptin was connected with the appearance of the drug resistance phenomenon to cisplatin, in cases of gastroesophageal adenocarcinomas. In turn, in an in vitro model, the exposition of the AGS Cis5 and OE33 cell lines to a leptin receptor antagonist resulted in the sensitization of the cells to the drug [12]. Leptin exerts its various activities through interacting with receptors, such as the leptin overlapping transcript (LEPROT), leptin receptor overlapping transcript-like 1 (LEPROTL1) and leptin receptor (LEPR), through which several signaling pathways are activated i.e., JAK/STAT, MAPK, PKC, JNK and PI3K/AKT pathways [13]. However, Saxena et al. show that the JAK/STAT signaling pathway is a key cascade associated with leptin activity [14].

Cisplatin, which has a therapeutic effect, conditioned by the induction of genetic material degradation on the molecular level, activating the proapoptotic pathways as well as oxidative stress, is one of the drugs used in cases of endometrial cancer [15]. Given the role of leptin in carcinogenesis [6,7,8,9,10], the development of drug resistance is connected with the expression of leptin [12]. To the best of our knowledge, so far, no study has been carried out to investigate the effect of cisplatin on leptin-related genes in an endometrial cancer cell line. The current research aimed to examine changes in the expression leptin and leptin-related genes depending on the concentration of leptin or cisplatin and exposure time of endometrial cancer cells to the drug or leptin. Additionally, we determined the cytotoxicity of cisplatin, by activating the apoptosis process in endometrial cancer cells.

## 2. Results

### 2.1. Cisplatin Cytotoxicity Assay

**Figure 1:**
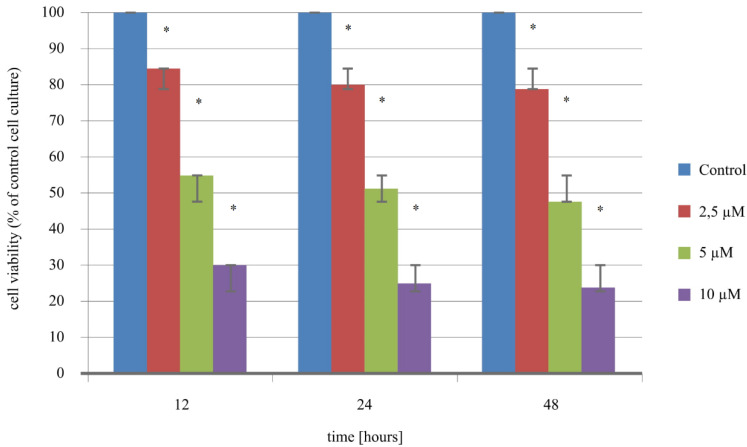
Outcomes of the Sulforhodamine B cytotoxicity assay (*—statistically significant differences in comparison to the control cell culture; *p* < 0.05).

The cytotoxicity analysis showed that regardless of the concentration of cisplatin added to the culture, the percentage of viable cells decreases compared to the control culture. The results showed that the lowest concentration of cisplatin causes an approximate decrease of 20% in viable cells. However, increasing the concentration of the drug to a cisplatin concentration of 5 µM can be considered the average inhibitory concentration (IC_50_) of the drug relative to the Ishikawa endometrial cancer cell line. In turn, when 10 µM of cisplatin was used, the percentage of viable cells was in the range of 23.33–30.01% (Figure 1). Differences in the percentage of viable cells under various conditions of the cell culture in comparison to the control culture were statistically significant (for 2.5. µM of cisplatin: H_12 vs. C *p* = 0.001; H_24 vs. C *p* = 0.001; H_48 vs. C *p* = 0.001; for 5 µM of cisplatin H_12 vs. C *p* < 0.00001; H_24 vs. C *p* = 0.0002; H_48 vs. C *p* < 0.00001; for 10 µM cisplatin H_12 vs. C *p* < 0.00001; H_24 vs. C *p* < 0.00001; H_48 vs. C *p* < 0.00001).

### 2.2. Leptin Cytotoxicity Assay

**Figure 2:**
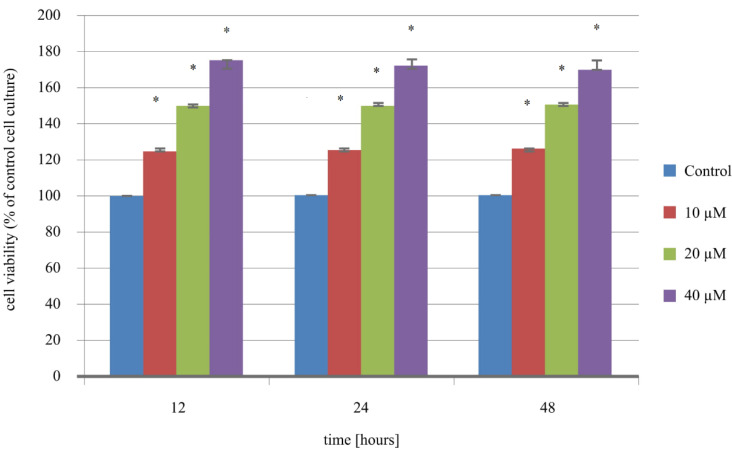
The results of increasing the concentration of leptin on endometrial cancer cell viability (*—statistically significant differences in comparison to the control cell culture; *p* < 0.05).

In turn, leptin added to the cell culture was associated with increasing the percentage of viable cells compared to untreated endometrial cancer cells (control). The analysis showed that regardless of leptin concentration, leptin promoted the proliferation of Ishikawa cells: treated cells with 10 ng/mL caused an increase of 24% in viable cells; for 20 ng/mL, an increase of approximately 49%; 40 ng/mL of leptin produced an increase in endometrial cancer cell viability of about 74% (Figure 2). The statistical analysis indicated statistically significant differences for 10 ng/mL of leptin: H_12 vs. C *p* = 0.001; H_24 vs. C *p* = 0.001; H_48 vs. C *p* = 0.001; for 20 ng/mL of leptin: H_12 vs. C *p* < 0.00001; H_24 vs. C *p* < 0.00001; H_48 vs. C *p* < 0.00001; and for 40 ng/mL of leptin H_12 vs. C *p* < 0.00001; H_24 vs. C *p* < 0.00001; H_48 vs. C *p* < 0.00001).

### 2.3. Morphology of Endometrial Cancer Cells Exposed to Cisplatin or/and Leptin

Assessing the changes in the cell morphology of the Ishikawa line, it can be seen that regardless of whether the cells were treated with cisplatin, leptin or a combination of thereof, the morphological changes of the cells were dependent on the concentration and exposure time. The higher the concentration and exposure time to cisplatin or leptin, the more pronounced the changes. After the use of cisplatin, it was observed that the round membranes of endometrial cancer cells take the shape of irregular polygons, their height is reduced by 1.3 µM + 0.02 µM and their surface roughness decreases. In turn, under the influence of leptin, an increase in cell size and discrete thickening of the cell membrane and greater surface roughness compared to the control was observed. Cisplatin has caused a limited reversal of changes.

### 2.4. Evaluation of Caspase-3, -8 and -9 in the Ishikawa Cell Line Treated with Cisplatin

**Figure 3:**
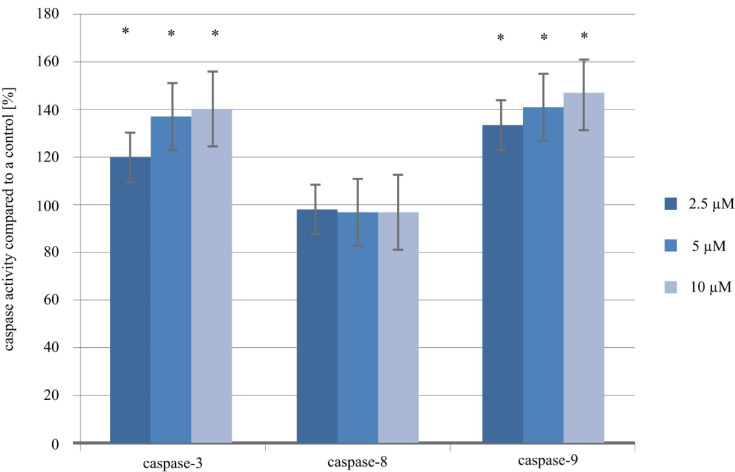
Caspase-3, -8 and -9 activity in the Ishikawa cell line exposed to 2.5 µM, 5 µM and 10 µM cisplatin for 24 h (*—statistically significant differences in comparison to the control cell culture; *p* < 0.05; control: cells treated with PBS; 100%).

**Figure 4:**
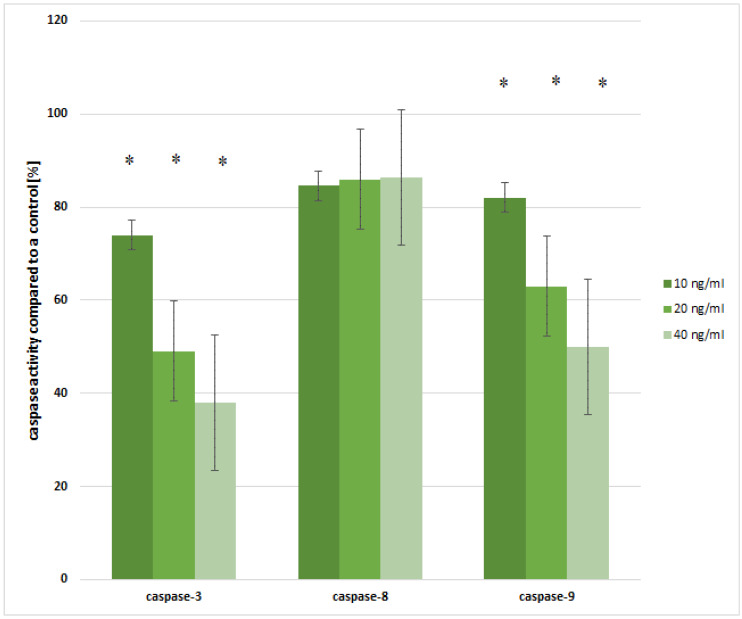
Caspase-3, -8 and -9 activity in the Ishikawa cell line exposed to 10 ng/mL, 20 ng/mL and 480 ng/mL of leptin for 24 h (*—statistically significant differences in comparison to the control cell culture; *p* < 0.05; control: cells treated with PBS; 100%).

To assess the proapoptotic properties of cisplatin and leptin, commercially available assays for the analysis of three caspases were used (Figure 3 and Figure 4). Differences in caspase-3 and -9 activity were statistically significant (*p* < 0.05). It could be observed that caspase-3 activity was higher in cells treated with cisplatin 20.01% (2.5 µM of cisplatin; *p* = 0.0000) to 40.09% (10 µM of cisplatin; *p* = 0.0000) compared with the control culture. Additionally, a higher caspase-9 activity, compared to the control, was observed in the endometrial cancer cell cultures exposed to cisplatin (2.5 µM of the drug = 133.58%; *p* = 0.0000; 5 µM = 140.55%; *p* = 0.0000; 10 µM = 147.09%; *p* = 0.0000). Moreover, analysis of caspase-8 activity did not indicate significant changes in all drug variations compared with an untreated cell control (98.03%, 97.09%, 97.08%; *p* > 0.05, Figure 3).

### 2.5. Evaluation of Caspase-3, -8 and -9 in the Ishikawa Cell Line Treated with Leptin

However, considering the effect of leptin on caspase-3, -8 and -9 activity, a decrease in their activities compared to the control cell culture was observed (*p* < 0.05). For caspase-3, the following activity pattern can be observed: 10 ng/mL of leptin gave a decrease of about 26% (*p* = 0.0021); 20 ng/mL gave a decrease by about 41% (*p* = 0.0000); and 40 ng/mL of leptin caused a decrease of approximately 62% in comparison to the control (*p* = 0.0000). Furthermore, for caspase-9, a decrease in its activity caused by 10 ng/mL of leptin was noticed by 18% (*p* = 0.0029), for 20 ng/mL of leptin by 37% (*p* = 0.0000) and for 40 ng/mL of leptin by 50%, compared to the control (*p* = 0.0000), while changes in caspase-8 activity were not statistically significant in the endometrial cancer cell line exposed to leptin in all leptin concentration variations (*p* > 0.05; Figure 4).

### 2.6. Expression Pattern of Leptin in Endometrial Cancer Cells Exposed to Cisplatin

**Figure 5:**
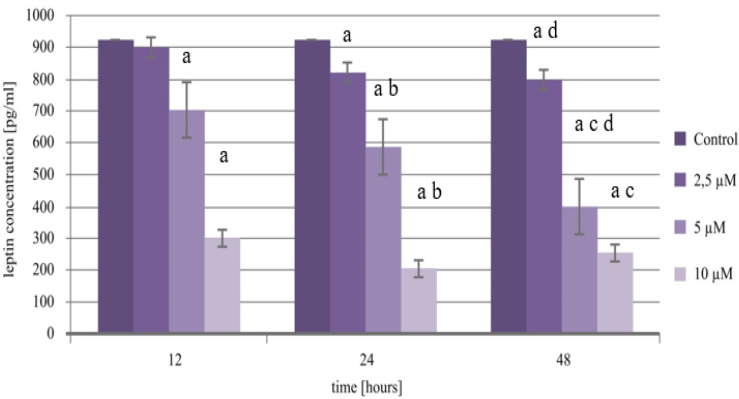
Changes in the expression of leptin in the endometrial cancer cell line treated with cisplatin, obtained by the ELISA assay (a—statistically significant differences in the expression of leptin between cells exposed to cisplatin vs. control *p* < 0.05; b—statistically significant differences in the expression of leptin between 12 vs. 24 h of exposition of cisplatin *p* < 0.05; c—statistically significant differences in the expression of leptin between 24 vs. 48 h of exposition of cisplatin *p* < 0.05; d—statistically significant differences in the expression of leptin between 12 vs. 48 h of exposition of cisplatin *p* < 0.05).

Based on the obtained results, changes in the level of the expression of leptin can be noticed, but in the culture not exposed to cisplatin, its level is higher than in the case of endometrial cancer cells incubated with the drug. This trend is noticeable not only on the mRNA level but also on the protein level. Cisplatin added to the culture with a concentration of 2.5 µM induced a decrease in leptin transcriptional activity irrespective of the exposure time. The protein level of leptin also had a gradual decline, with which the decrease in its concentration was less noticeable, which confirms that after only 24 h of incubation, it led to a statistically significant decrease in expression when compared to the control (*p* = 0.0000). Increasing the concentration of the drug to 5 µM caused a continued significant drop in the levels of leptin, with which, on the protein level, as far as the time of effect for cisplatin on the cells increased, this drop was on the level of about 120–185 pg/mL (*p* = 0.0000). Additionally, when using 10 µM of cisplatin for the exposition of endometrial cancer cells to a chemotherapy drug, the expression trend observed in lower concentrations is maintained. Nonetheless, however, it is worth noticing that a 48 h incubation led to an increase in the level of protein, but it was not significant statistically (*p* > 0.05). Changes in the expression profile of leptin in the Ishikawa cell line culture exposed to cisplatin are presented in Table 1 and Figure 5.

### 2.7. Expression Pattern of Leptin-Receptors in the Endometrial Cancer Cell Line Treated with Cisplatin

Analysis of changes in the level of leptin in endometrial cancer cells exposed to cisplatin at the protein level showed that the statistically significant differences were visible for cells treated with 2.5 µM of cisplatin after only 48 h in comparison to the control (*p* = 0.0031). Therefore, for the higher concentrations of cisplatin, regardless of the exposure time, changes in the level of leptin were always statistically significant when compared to the control cell culture and between exposure times (*p* < 0.05).

Furthermore, the results of the expression of leptin receptors in the Ishikawa cells exposed to 5 µM of cisplatin (average inhibitory concentration) obtained by the microarray and RTqPCR techniques were presented in Table 2.

Similar to the expression profile of leptin under cisplatin treatment, the analysis showed that the level of leptin receptors was downregulated in the cells treated with 5 µM of cisplatin, regardless of the exposure time. RTqPCR validated the expression pattern of analyzed genes observed in the microarray analysis.

### 2.8. Expression Pattern of JAK2 and STAT3 in Endometrial Cancer Cell Line Treated with Cisplatin

Next, we examined the variances in the mRNA and protein expression of JAK2 and STAT3 in the Ishikawa endometrial cancer cells treated with 5 µM of cisplatin and 10–40 ng/mL of leptin. The results showed that cisplatin reduces the transcriptional activity of selected genes, while leptin promoted the expression of mRNA *JAK2* and *STAT3* in endometrial cancer cells (Table 3; *p* < 0.05).

### 2.9. RNAi Analysis

**Figure 6:**
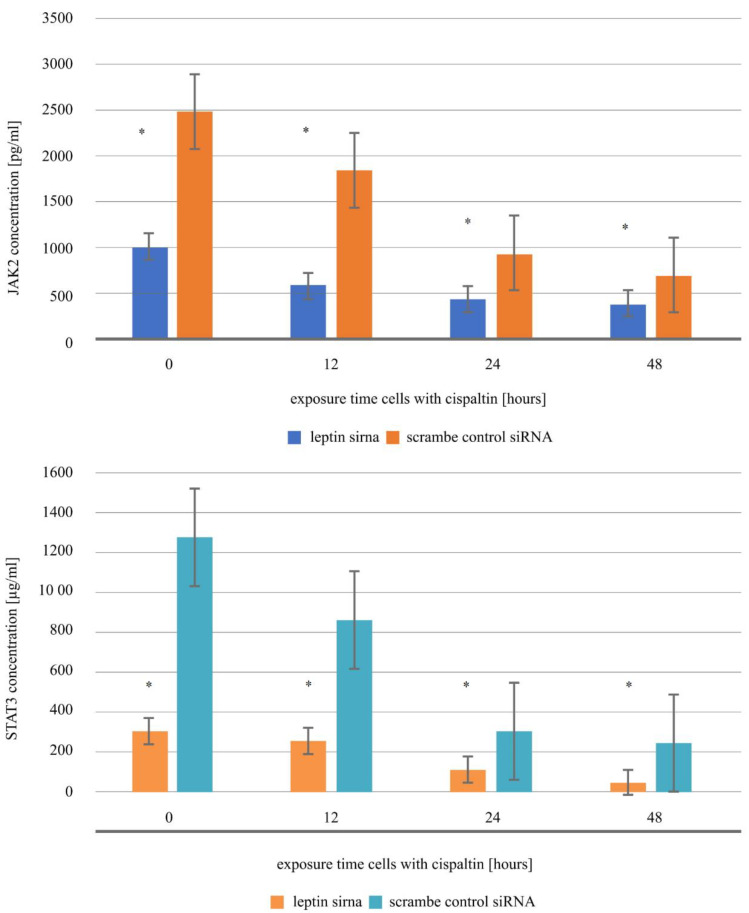
Leptin siRNA inhibited JAK2 and STAT3 gene expression in the Ishikawa endometrial cancer cell line treated with cisplatin and in the control culture (*—statistically significant differences *p* < 0.05).

The last step of our study was to examine the level of JAK-2 and STAT3 protein in the endometrial cells exposed to 5 µM of cisplatin and transfected with leptin siRNA or scramble control siRNA by the ELISA assay method. Cells incubated for 0 h with the drug constituted the control culture in this stage of molecular analysis. The obtained results showed that leptin siRNA inhibited the expression of JAK2 and STAT3 proteins both in the Ishikawa culture untreated with cisplatin (0 h) and the endometrial cancer culture incubated with cisplatin for 12, 24 and 48 h. Taking into account the expression pattern of the selected proteins in the culture with leptin siRNA and the culture with a scramble control siRNA (a negative control of the experiment), it can be observed that cisplatin intensifies the effect of leptin siRNA. Statistical analysis showed that regardless of the exposure time of the Ishikawa cell line with the siRNA vector, the variances in the expression profile of JAK2 and STAT3 were statistically significant compared to cells treated with the scramble control siRNA (Figure 6; *p* < 0.05).

## 3. Discussion

Gynecological tumors constitute 16% of all tumors and, simultaneously, are the cause of approximately 14% of deaths in women with cancer. In this group of tumors, a high frequency of malignant tumors can be distinguished, for example, cervical, endometrial and ovarian cancer, as well as relatively rare tumors of the fallopian tube, vagina, vulva and choroid cancer in gynecological and oncological practices [16]. Besides, attention is drawn to that in the situation of an occurrence of the neoplastic process, patients that are not overweight or obese have a better prognosis than patients with an improper body mass [17,18]. Moreover, in the case of endometrial cancer, it has been confirmed that there is an additional correlation between obesity and the risk of this cancer developing [19,20]. Other than this, attention is also brought to that the occurrence of overweight or obesity in childhood already constitutes a significant factor that increases the ability to induce the carcinogenic process within the endometrium [21]. Because of this, it also seems reasonable to come to know the molecular foundation that lies at the basis of overweight/ obesity, in which a key factor in maintaining the balance between energy and appetite is leptin [1,2], therefore cisplatin may influence the loss of body mass through affecting the expression of leptin during anticancer therapy. Our study confirmed the anticancer properties of cisplatin via the assessment of caspase activity and Sulforhodamine B sodium salt assay results. The obtained data suggest that 5 µM of cisplatin is the average inhibitory concentration and apoptosis is induced by cisplatin potentially via the mitochondrial-dependent pathway (increasing caspase-3 and -9 activities) [22]. Our observation of selected caspase activity is similar to the observation made by Ho et al. concerning tongue cancer treated with berberine [23]. Additionally, this study confirmed that cisplatin exerted its effect via the leptin pathway. The results of the changing levels of JAK2 and STAT3 proteins in cells treated with leptin siRNA compared to cells incubated with scramble siRNA (as a negative control) indicated that leptin siRNA and cisplatin exert a synergistic effect on the expression of assessed genes associated with the JAK/STAT signaling cascade. The obtained results in this stage of our work are in line with expectations because of the excessive activation of the JAK/STAT pathway observed in the course of endometrial cancer [24]. The effect of the influence of cisplatin via cascades activated by leptin was also confirmed by examining the changes in the expression of JAK2, STAT3, caspase-3,-8 and -9 in endometrial cell cultures exposed to leptin in three different concentrations [24,25]. That is said as the expression pattern of these selected genes in these two cell cultures was the opposite. This observation also showed the impact of leptin in promoting cell proliferation, the survival of endometrial cancer cells [26].

Uchikova et al. showed in their work that leptin is a key and incredibly important factor in the context of endometrial cancer [27]. As well as that, observations made by Daly-Brown et al. highlight the meaningfulness of the signals induced by leptin, as signal cascades are critical in the gaining of the ability for metastasis by cells that make up the mass of the tumor, which is reflected by a worse prognosis in patients with higher leptin levels [28]. Interesting observations are delivered by the study conducted by Gong et al., they incubated Ishikawa cell line cells, which are the same as we used in our study, with leptin at a concentration of 100 ng/mL. They concluded that the intensification of the proliferation of tumor cells indicated that this effect is probably caused by phosphorylation of extracellular kinase regulated by the ERK1/2 signal [29]. A similar conclusion was presented by Liu et al. who used a range of concentrations of leptin and incubation times with leptin of cells of the Ishikawa cell line. They indicated that depending on the dosage and time for the proliferation rate of endometrial cancer cells, as well as that the discussed adipokine has a substantial effect on the induction of cell metastasis in the Matrigel invasion test [30]. These observations highlight the importance of studying the changes in the level of leptin in answer to treatment with agents with proven anticancer action [12,27,28]. Based on our observations, it can be observed that under the influence of the addition of cisplatin to the endometrial cancer cells of the Ishikawa line, the expression of leptin is silenced, due to the fact, it seems that establishing oncological therapy of endometrial cancer on cisplatin is a reasonable action. Nonetheless, however, it is also worth taking into consideration that on the protein level, when using the lowest of the concentrations of cisplatin, a statistically significant change in the level of leptin in comparison to the culture unexposed to the drug was noted after only 48 h of cell incubation with the drug. This can be a result of several factors, whose effect can additionally overlap, which results in their synergistic action relative to the concentration of leptin. Firstly, it is possible that in the chosen model of the cell line cisplatin expresses its action with higher concentrations (5 µM, 10 µM), which is confirmed by the results obtained by us. Secondly, 2.5 µM of cisplatin is possibly not enough to affect the signaling pathways dependent on leptin relative to the short time (12 and 24 h), which is only extended to 48 h of cell incubation with the drug, causing changes in the induced signaling cascades. On the other hand, however, determining the lack of an answer from the cells, expressed by changes in the expression of leptin in answer to 2.5 µM of cisplatin is not fully justified. Because of this, it seems justified that differences in the mRNA level are expressed first. Additionally, the transcriptome is a system, which most likely reflects changes in the cellular environment, e.g., through drug introduction.

Considering the effect of cisplatin on the concentration of leptin relative to the dose and stimulation time of the cells that we noted, it is required not to forget that the correct level of leptin is required to keep an energetic balance on the correct level [1,2]. Therefore, leptin should be determined in vivo in patients with endometrial cancer before, during and after cisplatin treatment in the subsequent stages of the study. Because of this, it should also be remembered that even in the treatment of a described loss of an adequate answer to treatment with cisplatin, increasing its dosage is not the correct strategy of overcoming the occurring drug resistance [31]. This is key when you consider reports that a low level of leptin was diagnosed in oncological patients, in which additional disturbances were found such as anorexia [32,33,34]. Observations made by Yuan et al. indicate the need to analyze changes in leptin concentration in the context of endometrial cancer and therapy, they observed no statistically significant expression of leptin in a group of women with endometrial cancer compared to the control, but only in the situation when the seen level of leptin was normalized using the Body Mass Index (BMI) [35]. Matta et al. analyzed the concentration profile of leptin in a group of 80 patients with high grade serous epithelial ovarian carcinoma (HGSOC), who indicated resistance to platinum. In their study, they confirmed how important it is to find molecular markers that detect drug resistance. Firstly, because resistance to treatment with platinum can currently be determined based on phenotypic symptoms of the loss of an answer to treatment [36]. They observed that in the group of patients with median drug resistance (Me) concentrations of leptin marked through the ELISA method were 672 pg/mL vs. in patients without drug resistance Me = 724 pg/mL, the difference was not significant (*p* < 0.05). At the same time, they indicate that simultaneously marking leptin and a second marker CA-125 allows identifying patients who will have a resistance to platinum therapy [37].

Furthermore, molecular marker systems give the ability to display the occurrence of drug resistance before phenotypic changes [38,39], by which, therapy can be changed or adapted at an early enough stage, therefore minimizing the risk of cancer development. Taking into account our observation, our next studies ought to be focused on evaluating leptin and leptin-related pathways in both in vitro and in vivo models and assessing the influence of cisplatin in different endometrial cancer cell lines (EC-1-A, HEC-1-B corresponding to histological grade 2 (G2) and KLE corresponding to histological grade 3 (G3)). Therefore, future analysis ought to be focused on analyzing changes in the morphology of the Ishikawa endometrial cancer cell line under cisplatin and leptin treatment by using more precise methods, such as the DAPI assay. The research conducted by us in this work has clinical value, which should be included in the strengths of this work. First, such a comprehensive analysis of the effects of cisplatin on the expression of leptin-dependent genes in the endometrial cancer line was performed for the first time. Our results indicate how important of an issue education about proper nutritional supplements in patients with endometrial cancer and weight reduction is. This contributes to a faster and clearer response to cisplatin treatment. Additionally, it should be borne in mind that cisplatin has multidirectional activity at the molecular level, so the observed changes can be the result of many components. Besides, the results obtained by us can become a starting point for designing a personalized therapeutic strategy. Based on the concentration of leptin, history of the occurrence and the degree of overweight/obesity, the individually selected dose of cisplatin and the frequency of its administration can be predicted. Additionally, in-depth analyses of the morphology of endometrial cancer cells obtained from tissue collected for the histopathological examination will provide information, including on the condensation of genetic material in the cell nucleus and the extension of molecular analysis.

## 4. Materials and Methods

Our analysis consists of the following stages after treating cells with cisplatin and/or leptin:

Treatment of the Ishikawa endometrial cancer cell line with cisplatin or/and leptin in comparison to the control cell culture.

1. Analyzing the average inhibitory concentration (IC_50_) of cisplatin and leptin by performing the Sulforhodamine B assay.

2. Evaluating the apoptosis effect of cisplatin and leptin by evaluating caspase-3,-8 and -9 activities.

3. Assessment of the level of leptin at the transcriptome and proteome level in endometrial cancer cells exposed to cisplatin in comparison to the control culture.

4. Analyzing changes in the expression profile of leptin receptors in the endometrial cancer cell line treated with cisplatin (microarray and RTqPCR).

5. Determining the expression profile of leptin-related genes (JAK2 and STAT3) in the Ishikawa cell culture in different conditions.

6. Analyzing if cisplatin acts through the leptin signaling pathways by leptin knockdown (siRNA) and afterward examining the expression of key genes in these pathways.

### 4.1. Cell Culture

In this study, for each biological replicate, three technical replicates were performed. Endometrial cancer cells obtained from the Ishikawa cell line (European Collection of Authenticated Cell Cultures; ECACC 99040201) were exposed to three different concentration of cisplatin: 2.5 µM, 5 µM, 10 µM or three different concentrations of leptin: 10 ng/mL; 20 ng/mL; 40 ng/mL. The cells were incubated with the drug for the following times: 12; 24; and 48 h and compared to untreated cells which constituted the control. The Minimum Essential Medium (MEM; Catalog number: 51411C) was dedicated to this cell line, 2mM of glutamine, 1% Non-Essential Amino Acids (NEAA) and 5% Fetal Bovine Serum (FBS) were added according to the manufacturer’s protocol. The cells were incubated at a constant temperature of 37 °C with a 5% CO_2_ enriched atmosphere (Direct Heat CO_2_; Thermo Scientific, Waltham, MA, USA). All reagents were obtained from Sigma Aldrich, St Louis, MO, USA. After 24 h from the seeding of the cells in six-well plates, either cisplatin or leptin was added in individual concentrations to the cell culture.

### 4.2. Cytotoxicity Test

The first step was evaluating the cytotoxic properties of cisplatin or leptin through the Sulforhodamine B sodium salt assay (Sigma Aldrich, Merck, Catalog number 3520-42-1) according to the manufacturer’s protocol. The first stage was related to the preparation of cells for the experiment. For this purpose, endometrial cancer cells in the logarithmic growth phase were seeded into a 24-well plate in an amount of 20,000 cells/2 mL medium per well, followed by incubation at 37 °C, with a 5% CO_2_ enriched atmosphere for 24 h. In the second stage, the cells were incubated with cisplatin in the concentration range of 2.5 µM–10 µM or leptin in the concentration range 10 ng/mL-40 ng/mL for 12, 24 and 48 h. Untreated cells were the control. The absorption of solutions is determined spectrophotometrically at 490–530 nm. Based on the obtained absorbance readings at either a given cisplatin or leptin concentration and certain incubation time with it, the percentage of the value obtained for control cells (100%) was determined to be the calculated absorption value for individual cisplatin concentrations.

### 4.3. Induction of Apoptosis Activated via Analysis of Caspase-3, -8 and -9 Activity

Next, we assessed the influence of cisplatin or leptin on inducing apoptosis in the Ishikawa endometrial cancer cell line during cisplatin or leptin treatment by detecting caspase-3, -8 and -9 activity using the Caspase-8/Caspase-9 Colorimetric Assay Kit (R&D Systems. Minneapolis, USA, Caspase-8 Catalog number BF4100; Caspase-9 Catalog number K119-25) and EnzChek^®^ Caspase-3 Assay Kit #1 (Molecular Probes, Minneapolis, Willow Creek Rd. USA, Catalog Number E13183) assay according to the manufacturer’s protocol. The absorbance measurement at λ = 405 nm (caspase-8 and 9) and λ = 520 nm (caspase-3) permitted the level of caspase activity in cell lysates to be determined.

### 4.4. RNA Isolation

TRIzol reagent (Invitrogen Life Technologies, Carlsbad, CA, USA, Catalog Number 15596026) was used to isolate the total ribonucleic acid (RNA) from cell culture exposed to cisplatin and/or leptin and from the control Ishikawa endometrial cancer cell line as recommended in the manufacturer’s protocol. In the next stage, the RNeasy Mini Kit (QIAGEN, Hilden, Germany, Catalog Number 74104) and DNase I enzyme (Fermentas International Inc., Burlington, ON, Canada, Catalog Number 18047019) were used to purify extracts of RNA. Extracts were diluted in 20 µL of sterile water and frozen at −70 °C until the time when the molecular analysis was performed.

Next, the nucleic extracts were assessed by examining a fraction of 18S rRNA and 28S rRNA (1% agarose electrophoresis with 0.5 mg/mL ethidium bromide) and by evaluating RNA concentration (wavelength 260 nm) and purification (absorption ratio; wavelength 260 nm/280 nm; standard 1.8–2.0) through the use of spectrophotometry.

### 4.5. Expression of Leptin and Leptin-Related Genes in the Ishikawa Cell Line Treated with Cisplatin

The next part of molecular analysis in our work consisted of three stages. First, the microarray profile of leptin-related genes by using the HG-U 133_A2 microarray (Affymetrix, Santa Clara, CA and GeneChip™ 3’ IVT PLUS Reagent Kit and GeneChip™ HT 3’ IVT PLUS Reagent Kit (Thermo Fisher, Catalog Number 902416)) which was performed per the manufacturer’s instruction. Out of 22277 mRNA probes present on the microarray plate, 38 transcripts were related to leptin. The names of the probes were found on the Affymetrix NetAffx Analysis Center database after entering the query: “leptin” (http://www.affymetrix.com/analysis/index.affx; accessed on 20 January 2020). The Affymetrix Gene ArrayScanner 3000 7G and GeneChip^®^Command Console^®^Software were utilized for analysis of the fluorescence intensity. The detailed protocol that was used for microarray evaluation has been described by us previously [40].

Second, changes in the expression pattern of leptin (LEP) and its receptors, leptin receptor overlapping transcript (*LEPROT*), leptin receptor overlapping transcript-like 1 (*LEPROTL1*) and leptin receptor (*LEPR*) under the influence of cisplatin were evaluated. The Real-Time Quantitative Reverse Transcription Reaction (RTqPCR) with the use of the SensiFast SYBR No-ROX One-Step Kit (Bioline, London, UK) was used according to the manufacturer’s protocol. These genes were selected because of the differences in their expression, determined via the microarray technique, were the highest (Fold Change −5.0 ≤ FC ≥ 5.0). The effect of leptin on the JAK/STAT pathway was also determined and examined if cisplatin acts on JAK/STAT through this cascade, the expression of *JAK2* and *STAT3* were evaluated by the RTqPCR method.

The following stages of this reaction were indicated in the thermal profile: reverse transcription (45 °C for 10 min); activation of the polymerase (95 °C for 2 min); 40 cycles including denaturation (95 °C for 5 s); annealing (60 °C for 10 s); elongation (72 °C for 5 s). The primer sequence of selected genes evaluated via RTqPCR and their Probe Set ID on the HG-U 133_A2 microarray plate was presented in Table 4.

### 4.6. Level of Leptin and JAK 2 and STAT3 Obtained by ELISA Assay

The third part of the molecular examination was determining the expression of leptin on the protein level through the use of the ELISA assay alongside the Adiponectin Human solid-phase sandwich Enzyme-Linked Immunosorbent Assay ELISA KIT (Life Technologies Corporation, Invitrogen, USA; Catalog Number; KAC2281). The ELISA assay consists of the following stages: binding to the antigen; adding the detector antibody; addition of IgG HRP and TMB substrate solutions; and, finally, stopping the solution according to protocol. Next, the plate was read at 450 nm and a standard curve was generated to determine the concentration of leptin in the analyzed samples.

To assess if the drug exerts its effect via the leptin path in the cell culture exposed to cisplatin and leptin siRNA compared to the control culture, the levels of JAK-2 (JAK2 ELISA Kit ab253224) and STAT3 (STAT3 ELISA KIT ab126427) were evaluated via the ELISA assay.

### 4.7. RNA Interference

To evaluate the role of the leptin, the effect of leptin knockdown on Ishikawa cells, and to test whether cisplatin exerts its effect via leptin path, leptin siRNA was used.

The Ishikawa endometrial cancer cell line (exposed to 5 µM cisplatin as an average inhibitory concentration or without the drug) was transfected with leptin siRNA (sequence 5′-CCAAAUAUCCAACGACCUG-3′) or scramble control siRNA (Dharmacon, Lafayette, CO, USA) using Lipofectamine2000 reagent (Thermo Fisher Scientific, Lipofectamine™ 2000 Transfection Reagent; Catalog Number 11668027) according to the instruction in the manufacturer’s protocol. The culture was harvested 48 h after transfection.

### 4.8. Statistical Analysis

The licensed version of the Statistica 13.0 PL (StatSoft, Cracow, Poland) and the Transcriptome Analysis Console programs (Thermo Fisher Scientific) were used in the statistical analysis. First, the normality of the data distribution using the Shapiro–Wilk test (*p* < 0.05) was evaluated. Second, with the use of the ANOVA variance assay analysis, the differences shown were statistically significant, and next the post-hoc Tukey’s test was also conducted (*p* < 0.05).

## 5. Conclusions

Based on the obtained results, the effect of cisplatin on the expression profile of genes associated with the leptin-dependent signaling pathways (JAK2, STAT3) and changes in the expression of leptin itself and its receptors was confirmed. It was confirmed that cisplatin exerted its effect via the leptin pathway (RNAi). Determining leptin levels may appear to be one of the useful molecular markers for selecting a dose of cisplatin in endometrial cancer treatment and predicting treatment success. This procedure, in light of reports from other literature, is also an effective way of weakening the potential of metastasis of endometrial cancer cells [36]. The development of molecular marker systems is important because molecular changes appear ahead of phenotypic changes.

## Figures and Tables

**Table 1:** Changes in the level of mRNA and the protein of leptin on both concentration and time exposure of the Ishikawa cell line to cisplatin.

The Oncentration of Cisplatin (µM)	Time (h)	Microarray Data	RTqPCR	ELISA Assay Leptin (pg/mL)	
		FC	FC	Mean	Standard Deviation
**Control (untreated cells)**		-	-	921.977	0.345
	**12**	−4.96 ^a^	−5.12 ^a^	899.553	0.518
**2.5**	**24**	−7.89 ^a^	−7.99 ^a,b^	820.800 ^a^	0.381
	**48**	−9.01 ^a^	−8.54 ^a,c,d^	800.380 ^a,d^	0.532
	**12**	−11.69 ^a^	−12.03 ^a^	704.453 ^a^	0.402
**5**	**24**	−13.55 ^a^	−12.98 ^a,b^	585.997 ^a,b^	1.721
	**48**	−17.01 ^a^	−18.44 ^a,c,d^	401.317 ^a,c,d^	0.938
	**12**	−18.58 ^a^	−18.99 ^a^	300.209 ^a^	0.180
**10**	**24**	−19.03 ^a^	−19.30 ^a,b^	205.909 ^a,b^	0.992
	**48**	−21.48 ^a^	−21.78 ^a,c,d^	253.673 ^a,c^	1.773

a—Statistically significant differences in the expression of leptin between cells exposed to cisplatin vs. control *p* < 0.05; b—statistically significant differences in the expression of leptin between 12 vs. 24 h of exposition of cisplatin *p* < 0.05; c—statistically significant differences in the expression of leptin between 24 vs. 48 h of exposition of cisplatin *p* < 0.05; d—statistically significant differences in the expression of leptin between 12 vs. 48 h of exposition of cisplatin *p* < 0.05; (+) overexpression of gene (increased level of mRNAs); (-) suppressed gene expression (decreased level of mRNAs); FC—Fold Change; 12 h, 24 h, 48 h—periods of exposure to cisplatin.

**Table 2:** Changes in the expression pattern of leptin receptors in the endometrial cancer cell line treated with cisplatin (microarray and RTqPCR). Data presented as a Fold Change (FC) of gene expression in comparison to the control culture (*p* < 0.05).

Exposure Time Cells with 5 µM Cisplatin (h)	mRNA	ID	Microarray Data Fold Change	RTqPCR Data Fold Change
**12**	***LEPROT***	202377_at 202378_s_at	−5.82 * −5.36 *	−6.18 *
	***LEPROTL1***	202594_at 202595_s_at	−6.12 * −6.58 *	−6.77 *
	***LEPR***	209894_at 209959_at 211167_s_at 211354_s_at 211355_x_at 211356_x_at	−11.02 * −12.22 * −13.36 * −11.58 * −13.44 * −14.02 *	−12.09 *
**24**	***LEPROT***	202377_at 202378_s_at	−5.96 * −6.88 *	−7.04 *
	***LEPROTL1***	202594_at 202595_s_at	−7.55 * −8.02 *	−7.69 *
	***LEPR***	209894_at 209959_at 211167_s_at 211354_s_at 211355_x_at 211356_x_at	−14.33 * −14.02 * −11.02 * −12.08 * −14.01 * −14.77 *	−13.03 *
**48**	***LEPROT***	202377_at 202378_s_at	−8.11 −8.60	−9.58
	***LEPROTL1***	202594_at 202595_s_at	−9.02 −9.03	−9.14
	***LEPR***	209894_at 209959_at 211167_s_at 211354_s_at 211355_x_at 211356_x_at	−15.12 −15.73 −12.11 −13.08 −13.99 −13.02	−16.25

*—Statistically significant differences in comparison to the control culture (*p* < 0.05); (+) overexpression of gene (increased level of mRNAs); (−) suppressed gene expression (decreased level of mRNAs); ID—ID of the probe on a microarray; FC—Fold Change; 12 h, 24 h, 48 h—periods of exposure to cisplatin.

**Table 3:** Differences in the expression of mRNA *JAK2* and *STAT3* and protein in endometrial cancer cells treated with cisplatin and leptin in comparison with the control culture.

Group	Time (h)	mRNA	ID	Microarray Data Fold Change	RTqPCR Data Fold Change	ELISA (pg/mL)
**Control**				-	-	489
**Cells+ 5 µM cisplatin**	12	*JAK2*	205841_at	−1.74 ^a^	−1.79 ^a^	208 ^a^
	24			−1.71 ^a^	−1.41 ^a^	216 ^a^
	48			−1.81 ^a^	−1.77 ^a^	298 ^a^
**Cells+ 10 ng/mL of leptin**	12			+3.02 ^a^	+3.09 ^a^	1111 ^a^
	24			+3.14 ^a^	+3.11 ^a^	1196 ^a^
	48			+2.98 ^a^	+3.04 ^a^	1120 ^a^
**Cells+ 20 ng/mL of leptin**	12			+2.74 ^a^	+2.29 ^a^	987 ^a^
	24			+3.58 ^a^	+3.77 ^a^	1417 ^a^
	48			+3.47 ^a,c^	+3.68 ^a^	1402 ^a^
**Cells+ 40 ng/mL of leptin**	12			+4.02 ^a^	+4.14 ^a^	1854 ^a^
	24			+4.14 ^a^	+4.21 ^a^	1902 ^a^
	48			+4.84 ^a,c^	+4.77 ^a^	1869 ^a^
**Cells+ 5 µM cisplatin**	12	*STAT3*	208991_at	−2.03 ^a^	−2.36 ^a^	499
	24			−1.98 ^a^	−2.01 ^a^	454
	48			−1.47 ^a^	−1.51 ^a^	450
**Cells+ 10 ng/mL of leptin**	12			+2.07 ^a^	+2.14 ^a^	850 ^a^
	24			+2.11 ^a^	+2.19 ^a^	896 ^a^
	48			+2.36 ^a^	+2.41 ^a^	884 ^a^
**Cells+ 20 ng/mL of leptin**	12			+3.04 ^a^	+3.09 ^a^	914 ^a^
	24			+3.89 ^a^	+3.74 ^a^	948 ^a^
	48			+4.01 ^a,c^	+4.15 ^a^	1100 ^a^
**Cells+ 40 ng/mL of leptin**	12			+4.87 ^a,b^	+4.81 ^a^	1126 ^a^
	24			+4.22 ^a^	+4.23 ^a^	1161 ^a^
	48			+4.74 ^a^	+4.69 ^a^	1198 ^a^

a—Statistically significant differences in the expression of leptin between cells exposed to cisplatin vs. control *p* < 0.05; b—statistically significant differences in the expression of leptin between 24 vs. 48 h of exposition of cisplatin *p* < 0.05; c—statistically significant differences in the expression of leptin between 12 vs. 48 h of exposition of cisplatin *p* < 0.05; (+) overexpression of gene (increased level of mRNAs); (−) suppressed gene expression (decreased level of mRNAs); ID—ID of the probe on a microarray; FC—Fold Change; 12 h, 24 h, 48 h—periods of exposure to cisplatin.

**Table 4:** Nucleotide sequence of primers used in the RTqPCR reaction and their Probe Set ID on the HG-U 133_A2 microarray plate.

**mRNA**	**Probe Set ID on the Microarray Plate**	**Sequence**
**LEP**	207092_at	Forward GAAGACCACATCCACACACG Reverse AGCTCAGCCAGACCCATCTA
***LEPROT***	202377_at 202378_s_at	Forward GCTTGGAGAGGCAGATAACG Reverse AATGTCCTGGGTCCAGAGTG
***LEPROTL1***	202594_at 202595_s_at	Forward TGCAATGTGGGAAGAAATGA Reverse AAGGAGGAAGCAGAGGAAGG
***LEPR***	209894_at 209959_at 211167_s_at 211354_s_at 211355_x_at 211356_x_at	Forward ACAGTCCCTTTGTGGGTCAG Reverse TATCCGAGCTCCAGCGTACT
***JAK2***	205841_at	Forward AGTAAAAGTCCACCAGCGGA Reverse AGGAGGGGCGTTGATTTACA
***STAT3***	208991_at	Forward AAAGCAGCAAAGAAGGAGGC Reverse CTGGCCGACAATACTTTCCG
***ACTB***	-	Forward TCACCCACACTGTGCCCATCTACGA Reverse CAGCGGAACCGCTCATTGCCAATGG

Differences in transcriptional activity of selected genes obtained by RTqPCR were shown as a Fold Change of gene expression compared to the control (also known as the 2-∆∆Ct method) where: ∆∆Ct = ∆Ct (unknown test) —∆Ct (calibrator); ∆Ct (unknown sample) = Ct of the test gene—Ct of the reference gene; ∆Ct (calibrator) = Ct of the test gene - Ct of the reference gene. Result of relative expression: = 1—expression equal to control; >1—overexpression; <1—reduced expression.

## Abbreviations

BMI	Body mass index
ELISA	Enzyme-linked immunosorbent assay
HGSOC	High grade serious epithelial ovarian carcinoma
LEPR	Leptin receptor
LEPROT	Leptin receptor overlapping transcript
LEPROTL1	Leptin receptor overlapping transcript-like 1
mRNA	Messenger RNA
RTqPCR	Real-time quantitative reverse transcription reaction
siRNA	Small interfering RNA
